# Short (16-mer) locked nucleic acid splice-switching oligonucleotides restore dystrophin production in Duchenne Muscular Dystrophy myotubes

**DOI:** 10.1371/journal.pone.0181065

**Published:** 2017-07-24

**Authors:** Vanessa Borges Pires, Ricardo Simões, Kamel Mamchaoui, Célia Carvalho, Maria Carmo-Fonseca

**Affiliations:** 1 Instituto de Medicina Molecular, Faculdade de Medicina, Universidade de Lisboa, Lisboa, Portugal; 2 Center for Research in Myology, INSERM UMRS974, CNRS FRE3617, Sorbonne Universités, UPMC Univ Paris 06, Paris, France; International Centre for Genetic Engineering and Biotechnology, ITALY

## Abstract

Splice-switching antisense oligonucleotides (SSOs) offer great potential for RNA-targeting therapies, and two SSO drugs have been recently approved for treating Duchenne Muscular Dystrophy (DMD) and Spinal Muscular Atrophy (SMA). Despite promising results, new developments are still needed for more efficient chemistries and delivery systems. Locked nucleic acid (LNA) is a chemically modified nucleic acid that presents several attractive properties, such as high melting temperature when bound to RNA, potent biological activity, high stability and low toxicity in vivo. Here, we designed a series of LNA-based SSOs complementary to two sequences of the human dystrophin exon 51 that are most evolutionary conserved and evaluated their ability to induce exon skipping upon transfection into myoblasts derived from a DMD patient. We show that 16-mers with 60% of LNA modification efficiently induce exon skipping and restore synthesis of a truncated dystrophin isoform that localizes to the plasma membrane of patient-derived myotubes differentiated in culture. In sum, this study underscores the value of short LNA-modified SSOs for therapeutic applications.

## Introduction

Antisense oligonucleotides are powerful tools to modulate gene expression. Namely, antisense oligonucleotides can be used to induce RNA interference or RNase H based mechanisms of gene down-regulation, inhibit the function of microRNAs, or modulate splicing (for a recent review see [1]). Despite early clinical trial failures, new developments are fueling optimism in the antisense field. In particular, the design of novel chemical modifications and delivery systems is improving the potency and efficacy of these drugs in RNA-targeting therapeutic applications [1]. Recently, antisense oligonucleotide drugs have been approved for treating Duchenne Muscular Dystrophy (DMD) [2] and Spinal Muscular Atrophy [3].

DMD was the first disease shown to benefit from antisense oligonucleotides designed to modulate pre-mRNA splicing. This X-linked genetic disease is mainly caused by frame-shifting deletions or nonsense mutations in the *DMD* gene that result in a loss of functional dystrophin protein [4], leading to fatal progressive muscle wasting [5]. A related but milder form of the disease called Becker Muscular Dystrophy (BMD) is caused by in-frame mutations in the *DMD* gene that allow expression of an internally truncated but partially functional protein [6]. Antisense oligonucleotides have been extensively used to induce skipping of the exon containing a frame-shift deletion or a nonsense mutation, thus restoring the mRNA reading frame and producing an internally deleted protein similar to that observed in BMD patients. Due to mutation clustering in hotspot regions, the largest fraction of patients (approximately 13%) would benefit from skipping of exon 51 directed therapy [7].

To date, two distinct splice-switching antisense oligonucleotides (SSOs) complementary to the human dystrophin exon 51 sequence have been tested in clinical trials: drisapersen (Prosensa/GlaxoSmithKlein, presently BioMarin) and eteplirsen (Sarepta Therapeutics). Drisapersen is a 24-mer based on phosphorothioated 2’-O-methyl RNA chemistry and eteplirsen is a 30-nucleotide phosphorodiamidate morpholino. The promising results obtained prompted the accelerated FDA approval of Eteplirsen (Exondys 51). However, the levels of dystrophin restoration reported in these trials are still low [8–13]. Thus, much attention is devoted to finding new strategies that might improve the pharmacological properties of SSOs.

An alternative chemical modification with attractive properties for clinical applications is locked nucleic acid (LNA) [14]. LNA is a nucleotide analog carrying an altered ribose in which a methylene bridge connects the 2'-*O* with the 4'-*C* atoms in the furanose ring [15,16]. This bridge enables LNA to form a strictly *N*-type conformation that enhances the binding affinity against complementary RNA [17–19]. Several studies have shown that introducing LNA into antisense oligonucleotides is advantageous for a variety of gene silencing techniques [20,21] and recent clinical trials with an LNA-modified oligonucleotide targeting miRNA-122 (miravirsen) suggest that this may be an effective and safe strategy for patients chronically infected with hepatitis C virus [22]. LNA-based SSOs have also been tested in cellular and mouse models with encouraging results [23–28]. Inspired by a recent study that systematically addressed the optimal design of LNA-based SSOs targeting the human dystrophin exon 58 sequence [29], here we designed a series of LNA-modified antisense oligonucleotides and evaluated their ability to induce skipping of the dystrophin exon 51 in myoblast cells derived from a DMD patient.

## Materials and methods

### Databases

The sequence of *DMD* gene orthologues was analyzed on Ensembl Genome Browser [30]. Sequence alignment was performed on CLUSTALW (GenomeNet). UCSC Genome Browser [31] was used for visualization of the human *DMD* gene.

### Oligonucleotides

Splice-switching antisense oligonucleotides (SSOs) that contain a fully phosphorothioate modified backbone and 60% LNA, with two LNA-modified nucleotides at the 3’-end and one LNA-modified nucleotide at the 5’-end, were purchased from Exiqon (Vedbaek, Denmark). The AO51 2’-O-methyl-phosphorothioate SSO was gently provided by C. Trollet [32].

### Cell lines

Immortalized myoblasts derived from a DMD patient (DM8036) and from a control individual (KM155) were previously described [32]. Cells were maintained in Skeletal Muscle Cell Growth Medium (PromoCell) in 5% CO_2_, at maximum confluence of 60–70%. To induce differentiation, cells were plated at a density intended to reach 80% confluence 24 hours later, and then the medium was switched to differentiation medium: DMEM high glucose (Gibco) supplemented with ITS—Insulin, Transferrin, and Sodium Selenite (Sigma). To maintain cells differentiating, half of the differentiation medium was replaced each 2–3 days.

### Cell transfection

SSOs were transfected using Lipofectamine RNAimax Reagent (Invitrogen) according to the manufacturer’s instructions. Specifically, for a cell culture area of 1.9 cm^2^, 1 μL of Lipofectamine RNAimax in 15 μL of OptiMEM (Gibco) was added to 15 μL of SSO diluted in OptiMEM. For mock transfection, no SSO was used. Transfection was performed either simultaneously with myoblast platting or 3 days after initiating differentiation, as indicated on results.

### RNA isolation and reverse transcription-PCR

Total RNA was extracted using PureZol RNA isolation reagent (BioRad) and purified using DNAse I treatment (Roche) and acidic phenol extraction with UltraPure^TM^ Phenol:Cloroform:Isoamyl Alcohol (25:24:1, v/v; Invitrogen) according to the manufacturer’s instructions. Purity and quantity of purified RNA was analyzed using Nanodrop2000 (ThermoScientific). For cDNA synthesis, 0.6 μg of RNA was retrotranscribed in a 20 μL reaction using Transcriptor High Fidelity cDNA Synthesis Kit (Roche) and 20 pmol of reverse primer *DMD_*exon_54 (5'-GGAGAAGTTTCAGGGCCAAG-3'), at 55^°^C for 90 min. 0.5μL of cDNA was PCR-amplified in a 10 μL reaction with KAPA2G^TM^ Fast (Kapa Biosystems), with the primers *DMD_*exon_47F (5'-ACCCGTGCTTGTAAGTGCTC-3') and *DMD_exon_*53R (5'-TGACTCAAGCTTGGCTCTGG-3'). The cycling conditions were 95^°^C, 5 min, followed by 45 cycles of 95^°^C, 15 sec; 58^°^C, 30 sec and 72^°^C, 15 sec. PCR products were separated by electrophoresis on a 2% agarose gel followed by imaging on Chemidoc XRS^+^ system (BioRad) and band intensity quantification using the Image Lab 5.2 software (BioRad). For statistics and EC_50_ determination, Graphpad Prism 6 (GraphPad Software) was used.

### Protein extraction and Western blot

Protein extraction and Western blot methods were adapted from previous descriptions [33,34]. Cells on 3.8 cm^2^ culture plates were washed briefly with phosphate-buffered saline (PBS) at room temperature and then lysed in 40 μL of a buffer containing 0.1 M Tris-HCl pH 6.8 and 20% sodium dodecyl sulfate (SDS). Protein concentration was determined using the Pierce^TM^ BCA Protein Assay kit (ThermoScientific) according to the manufacturer’s instructions. Before SDS-PAGE, protein samples were treated with 1% Benzonase (Sigma), 14mM MgCl_2_ for 15 min, the homogenate was completed to a final composition of 75 mM Tris-HCl pH 6.8, 15% sodium dodecyl sulfate, 5% dithiothreitol, 20% glycerol and 0.01% bromophenol blue, and then boiled for 5 min. For Western Blot analysis, 1 μg of protein extract was loaded on a 7% polyacrylamide gel and run for 90 min (10 min at 60 V + 80 min at 100 V), with Precision Plus Protein Standards (BioRad) as molecular weight marker. Transfer buffer (0.6% Tris, 2.88% glycine, 0.01%SDS, 4% methanol) was used to transfer proteins to nitrocellulose BA85 membranes (Whatman Protran) with a Tank Transfer System (BioRad Mini Trans-Blot R Cell) for 90 min at 300 mA. Blots were first incubated with 5% non-fat dried milk in Tris-buffered saline containing 0.05% Tween-20 (TBST), for 1 hour, and then incubated overnight at 4^°^C with rabbit polyclonal anti-dystrophin antibody (Abcam ab85302, diluted 1:1000 in 5% non-fat dried milk in TBST). Secondary antibody Goat Anti-Rabbit IgG (H+L)-HRP (BioRad, dilution 1:3000), was incubated for 60 min at room temperature. The polyclonal antibody rabbit anti-Lamin A/C (Santa Cruz) was used as a loading control (overnight incubation at 4^°^C; dilution 1:10000). WesternBright Quantum (Advansta) was used to chemiluminescent detection and imaging with the Chemidoc XRS^+^ system (BioRad).

### Immunofluorescence microscopy

For microscopy analysis, cells were cultured onto 0.1% gelatin-coated glass coverslips (10x10 mm^2^, Normax). Cells were first fixed with 3.7% formaldehyde (freshly prepared from paraformaldehyde) in phosphate-buffered saline (PBS) for 10 min at room temperature, and then permeabilized with 0.5%Triton X-100 in PBS for 10 min at room temperature. Next, cells were incubated for 30 min at room temperature in 1%BSA and 0.05%Tween20 in PBS. Cells were then incubated with polyclonal rabbit anti-dystrophin antibody (Abcam ab85302, dilution 1:100) for 60 min at room temperature followed by incubation with tetramethylrhodamine (TRITC) conjugated donkey anti-rabbit antibody (Jackson ImmunoResearch; dilution 1:200) for 60 min. Antibodies were diluted in 1%BSA, 0.05%Tween20 in PBS. To counterstain the nuclei, cells were incubated for 10 min with 1 μg/mL 4’,6-diamidino-2-phenylindole (DAPI; Sigma). VECTASHIELD® Antifade Mounting medium (Vector Laboratories) was used. Cells were imaged with a LSM 710 Confocal Point-Scanning Microscope (ZEISS), using the lasers Diode 405–30 (405 nm) and DPSS 561–10 (561 nm).

## Results

**Fig 1:**
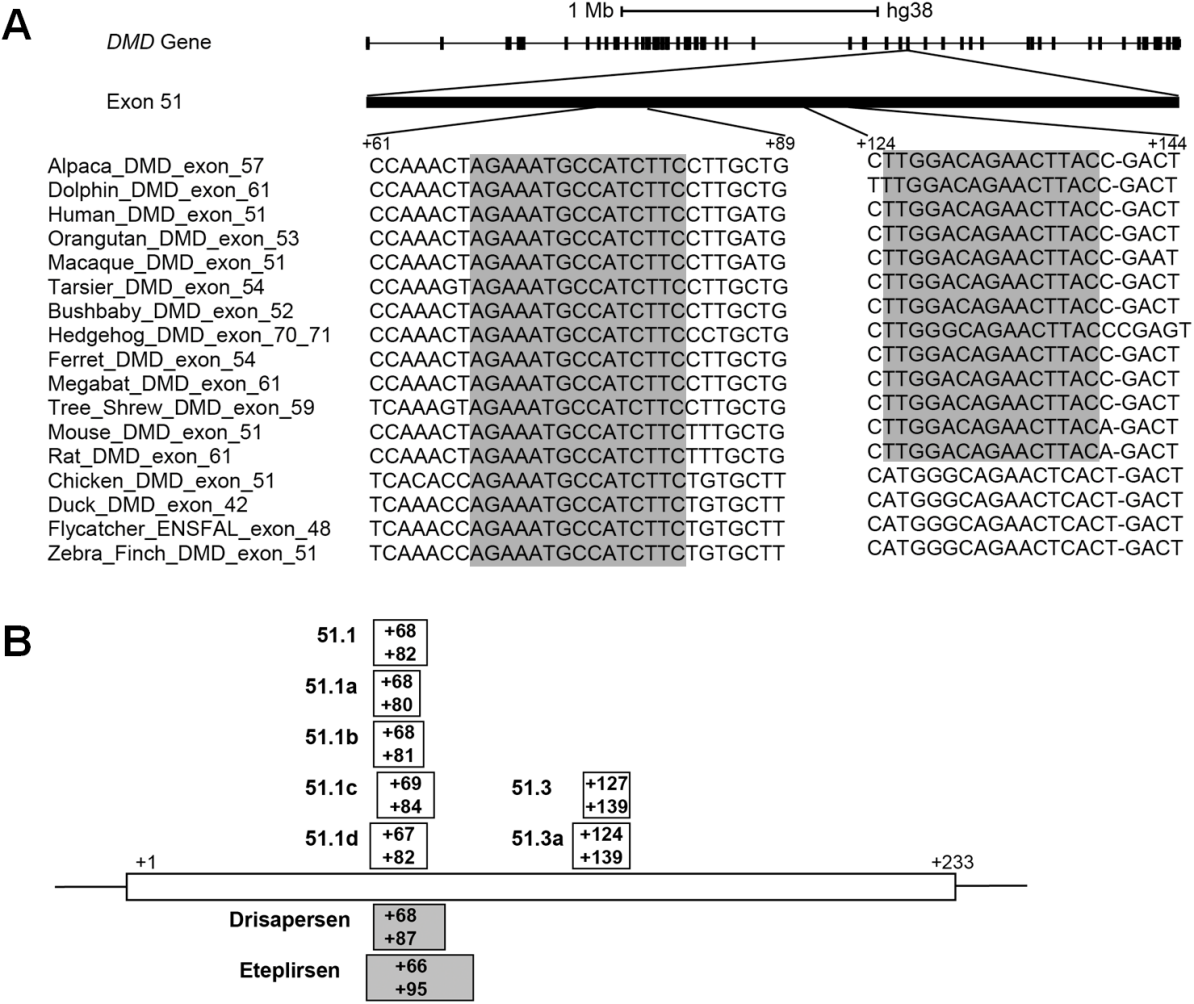
Oligonucleotide design. (A) Schematic representation of the human dystrophin gene and sequence alignment of two exon 51 regions across the indicated species. Conserved nucleotide sequences are shadowed. (B) Annealing sites of SSOs designed in this study are indicated by boxes. For comparison, the annealing sites of Eteplirsen and Drisapersen are indicated by shaded boxes. Position +1 corresponds to the first nucleotide in exon 51.

The rational design of an exon skipping oligonucleotide involves the selection of an antisense sequence targeting the exon that is to be skipped. Several parameters influence SSO activity, including the melting temperature, guanine-cytosine content, length of the oligonucleotide, and secondary structures or sequence motifs that correspond to splicing signals of the target RNA [35–38]. Here, we explored sequence conservation as a complementary strategy to design SSOs. A search for the longest evolutionary conserved stretches of DNA that are unique to exon 51 of the human *DMD* gene revealed two regions. One region contains a sequence of 15 nucleotides (from +68 to +82; Fig 1A) that is conserved across mammalian species ranging from primates (Human, Orangutan, Macaque, Tarsier, Bushbaby), to Tree-Shrew, Megabat, Hedgehog, Alpaca, Dolphin, Ferret, and rodents (Mouse and Rat), as well as bird species such as Chicken, Duck, Flycatcher, and Zebra-finch. Noteworthy, this highly conserved sequence is included in the region targeted by the drisapersen and eteplirsen SSOs that have been used in clinical trials. To investigate the influence of oligonucleotide length on the ability to induce exon skipping, we synthesized a 15-mer SSO targeting the conserved sequence (51.1), and additional 13-, 14- and 16-mers SSOs shifted by one or two nucleotides (Fig 1B). Another region contains a sequence of 15 nucleotides (from +125 to +139; Fig 1A) conserved across the 13 mammalian species analyzed. We synthesized a 13-mer SSO targeting this sequence (51.3), and an additional 16-mer SSO shifted by three nucleotides (Fig 1B).

To evaluate whether these SSOs modulate the splicing of endogenous human dystrophin pre-mRNA, we used myogenic cells derived from a DMD patient carrying a deletion of exons 48–50 that would be frame-corrected by skipping exon 51 [32]. Cultured myoblasts were transfected with each SSO and one day after transfection the cells were induced to differentiate into myotubes.

**Fig 2:**
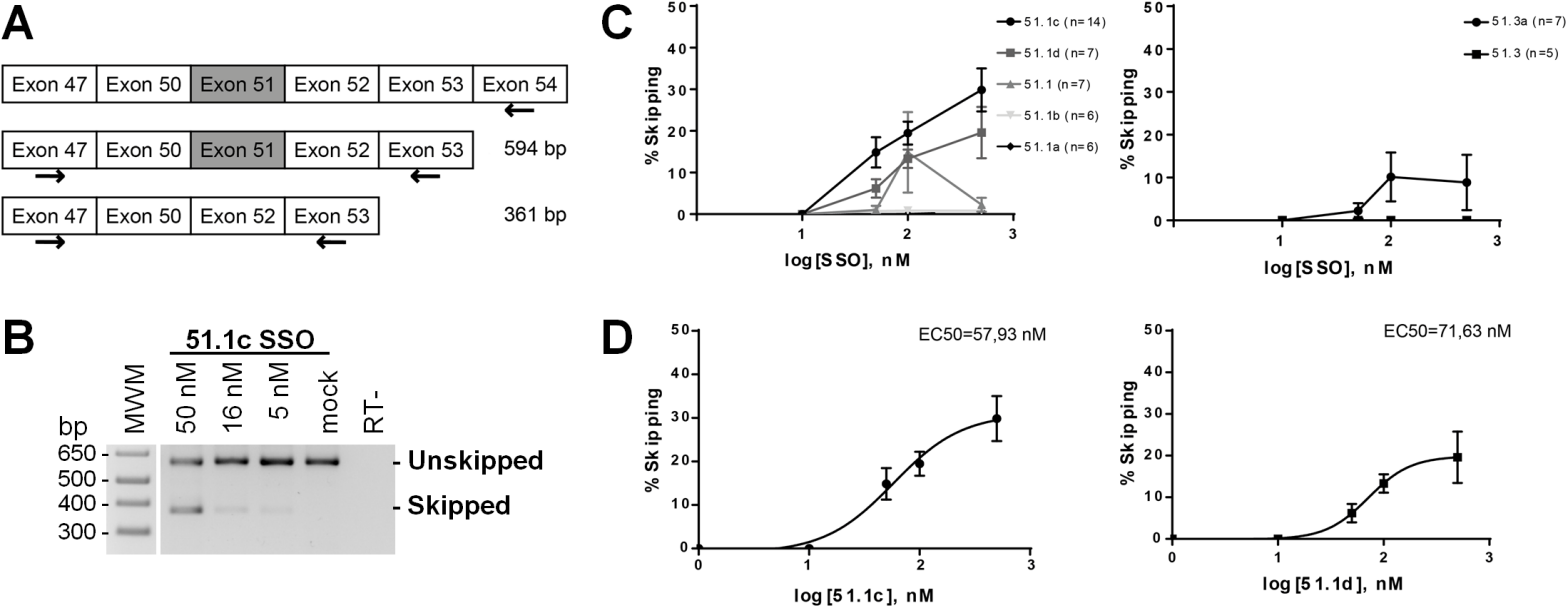
RT-PCR analysis of exon 51 skipping. (A) Schematic representation of *DMD* exons with the location of primers used for retrotranscription and RT-PCR analysis of exon 51 skipping; expected sizes of PCR products are indicated. (B) DM8036 cells were transfected with SSO 51.1c at the indicated concentrations and analyzed 2 days after induction of myotube differentiation. Electrophoresis of PCR products in agarose gels shows non-skipped and skipped transcripts. Mock: mock transfection; -RT: no retrotranscriptase. (C) Percentage of exon skipping induced by each SSO at day 7 post-induction of myotube differentiation. Values were obtained from quantification of the gels shown in S1 Fig and represent mean ± SEM. (D) Dose-response curves of the two most efficient SSOs. EC50: half maximal effective concentration.

For splice switching evaluation, transcripts were analyzed by RT-PCR. RNA was extracted from differentiated cultures, retrotranscription was performed with a primer specific for *DMD* exon 54 (Fig 2A) and PCR reactions with forward and reverse primers on exons 47 and 53, respectively (Fig 2A). In mock transfected cells, we observed a single band of approximately 600 bp that corresponds to *DMD* transcripts including exon 51 (Fig 2B). In cells transfected with SSO 51.1c, we detected a second band of approximately 360 bp that corresponds to *DMD* transcripts excluding exon 51 (Fig 2B). In order to assess the efficiency of exon skipping induced by each SSO, we quantified the relative intensity of the bands corresponding to skipped and unskipped transcripts observed in agarose gels from independent experiments (S1 Fig). Skipping percentage was calculated as [*skipped transcript*/(*skipped* + *unskipped transcripts*)] *x* 100. The skipping activity of the 16-mer 51.1c ranged between 15 and 25% in a concentration-dependent manner (Fig 2C). Shifting the SSO sequence by only two nucleotides (16-mer 51.1d) resulted in slightly lower efficiency while shorter SSOs (15-mer 51.1; 13-mer 51.1a; and 14-mer 51.1b) had very little (<10%) skipping activity (Fig 2C). The 13-mer 51.3 also presented very little skipping activity, whereas the 16-mer 51.3a targeting the same region had an efficiency in the range of 15% (Fig 2C). For SSOs 51.1c and 51.1d, a dose-response curve was generated taking into account the skipping percentage observed after transfection of different oligonucleotide concentrations. From these curves we estimated values for the half maximal effective concentration (EC50) in the range of 50 to 75 nM (Fig 2D).

We also observed that the skipping activity tended to be higher in cells analyzed 3 days after transfection (Fig 2B) than in cells analyzed 8 days post-transfection (S1 Fig). Since cells induced to differentiate no longer divide, it is unlikely that this effect is due to dilution of the oligonucleotides in daughter cells. One possibility is that either the transfection procedure or the presence of SSOs interferes with the differentiation process and/or cell viability, and therefore long-term differentiated cultures become enriched in non-modified cells. Another possibility is that SSOs are not efficiently recycled after annealing to nascent pre-mRNAs.

**Fig 3:**
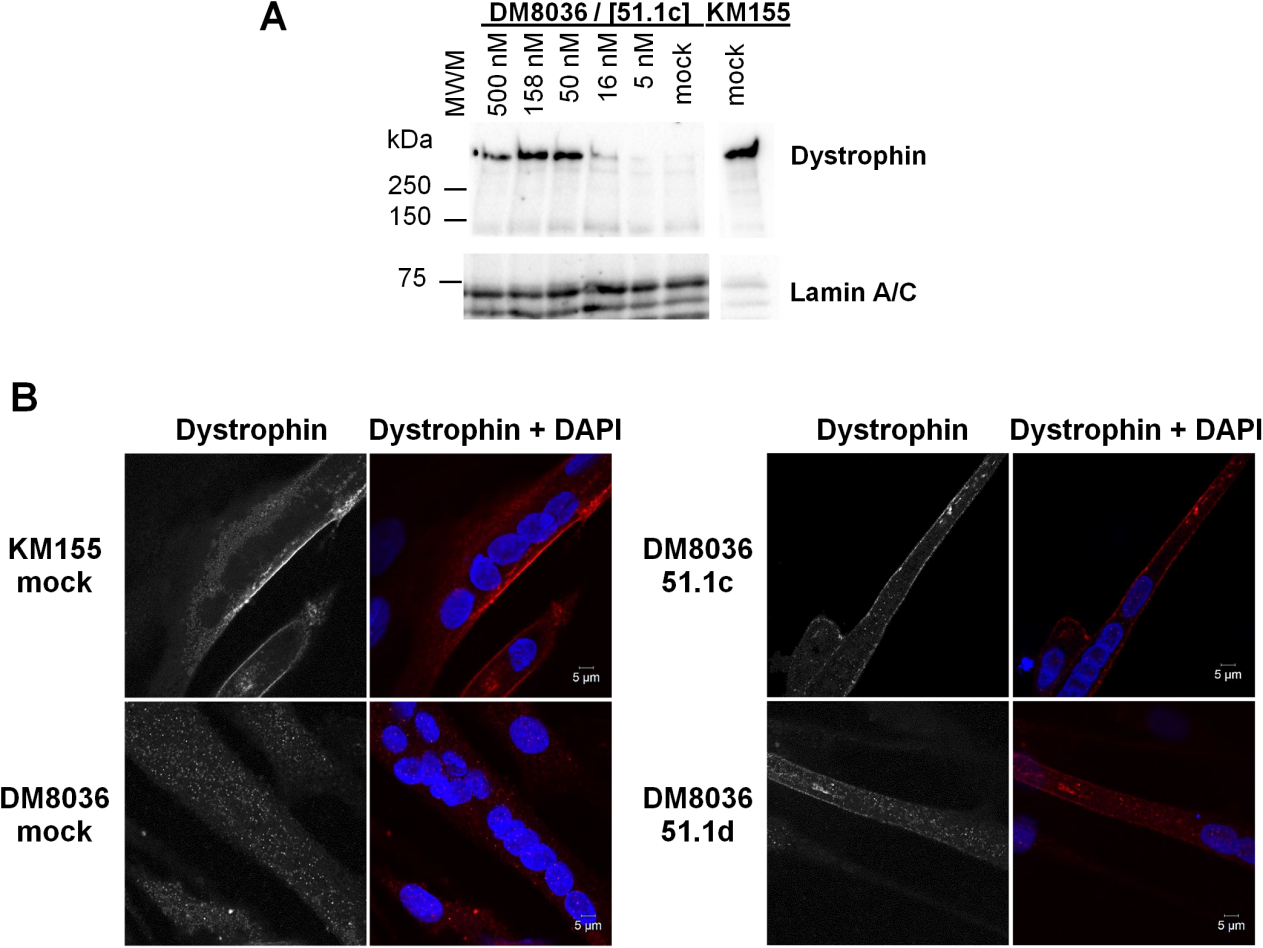
Analysis of dystrophin protein restoration. (A) Western blot analysis of total proteins extracted from cells derived from a healthy individual (KM155) and a DMD patient (DM8036). Patient myoblasts were either transfected with SSO 51.1c at the indicated concentrations or mock transfected. Western blotting was carried out at day 2 of differentiation using antibodies to dystrophin and Lamin A/C. The position of molecular weight markers (MWM) is indicated. (B) Immunofluorescence analysis of cells derived from a healthy individual (KM155) and a DMD patient (DM8036) at day 7 of differentiation using anti-dystrophin antibody (red staining). Nuclei were counterstained with DAPI (blue staining). Patient myoblasts were either transfected with the indicated SSOs at 50 nM or mock transfected.

In order to evaluate dystrophin protein synthesis restoration by LNA-SSO 51.1c, we used Western blot analysis. In control cells from a healthy individual (KM155), Western blot analysis with an anti-dystrophin antibody revealed a single high molecular weight band (Fig 3A) consistent with the size of wild-type dystrophin (427 kDa). As expected, no dystrophin band was observed in mock transfected patient DM8036 cells (Fig 3A). Transfection with LNA-SSO 51.1c resulted in the appearance of a band corresponding to truncated dystrophin protein with an estimated size of approximately 400 kDa (Fig 3A).

The localization of the rescued truncated protein in differentiated myotubes was assessed by immunofluorescence with anti-dystrophin antibody (Fig 3B). The vast majority of myotubes derived from KM155 cells were clearly labeled at the plasma membrane (Fig 3B), consistent with the well-established dystrophin localization at the cytoplasmic face of the muscle cell plasma membrane, or sarcolemma [39]. In contrast, practically all myotubes derived from patient DM8036 cells did not show any dystrophin labeling (Fig 3B). Upon transfection of LNA-SSOs 51.1c and 51.1d (Fig 3B), dystrophin labeling at the plasma membrane was observed in a subset of myotubes derived from DM8036 cells.

**Fig 4:**
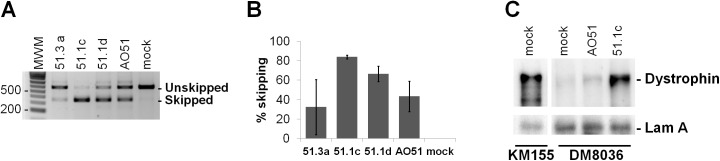
Comparison of LNA-SSOs and AO51. (A) DM8036 myoblasts were transfected with the indicated SSOs at 50nM and analyzed at day 2 of differentiation. Electrophoresis of PCR products in agarose gels shows non-skipped and skipped transcripts. Mock: mock transfection. (B) Percentage of exon skipping induced by each SSO. Values were obtained from quantification of gels (shown in A and S2 Fig) and represent mean ± SD (N = 4). (C) Western blot analysis of total proteins extracted from cells derived from a healthy individual (KM155) and a DMD patient (DM8036). Patient myoblasts were transfected with the indicated SSOs at 50 nM and induced to differentiate. Western blotting was carried out at day 5 of differentiation using antibodies to dystrophin and Lamin A/C.

Next, we compared the results obtained with short LNA-modified SSOs with a previously described oligonucleotide that carries full-length 2'-*O*-methyl-substituted ribose molecules and phosphorothioate internucleotide linkages, AO51 [32]. This oligonucleotide is also known as h51AON1 [40] and PRO051 [41]. Following successful results obtained in preclinical studies [42] and clinical trials [41,43], this oligonucleotide has been developed as an experimental drug termed drisapersen. However, the FDA rejected drisapersen in early 2016, due to safety issues [2]. As shown in Fig 4 and S2 Fig, the 16-mer LNA-modified SSO 51.1c is more effective than AO51 in inducing exon skipping and restoring synthesis of a truncated dystrophin isoform. We further observed that upon transfection into myoblasts both oligonucleotides have a similar effect on myotube differentiation (S3 Fig).

**Fig 5:**
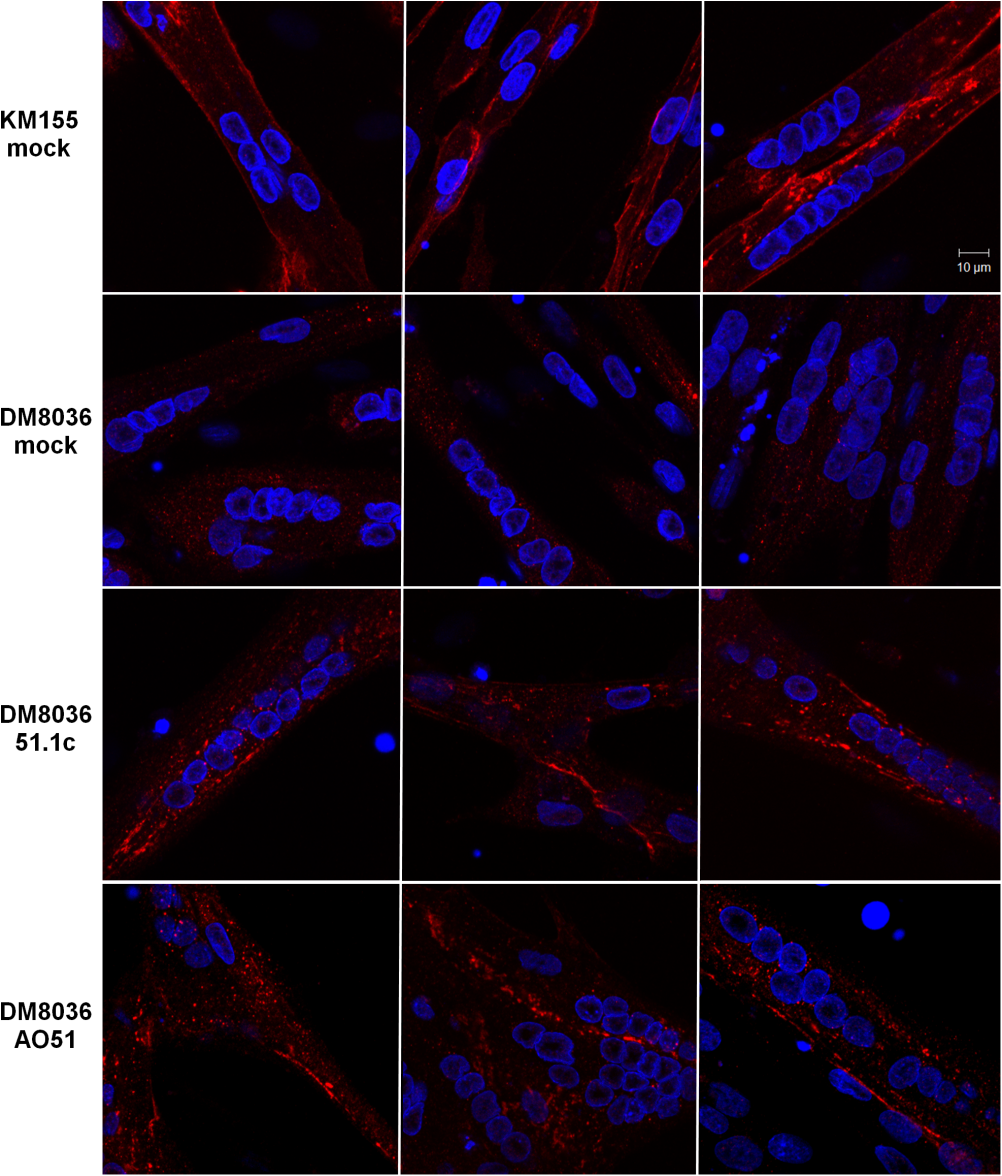
Dystrophin rescue in myotubes. DM8036 and KM155 myoblasts were induced to differentiate for 3 days. Myotubes were then either mock transfected or transfected with the indicated SSOs at 50 nM and fixed 2 days later. Immunofluorescence was carried out with anti-dystrophin antibody (red staining) and nuclei are highlighted with DAPI (blue staining).

Finally, we tested whether LNA-SSO 51.1c can rescue dystrophin production in differentiated myotubes. DM8036 myoblasts were first induced to differentiate for 3 days prior to SSO transfection. As shown in S3 Fig, approximately 70% of all nuclei observed in cultures that were not transfected with SSOs and were induced to differentiate for 3 days are present in multinucleated myotubes indicating that under these culture conditions the majority of myoblasts differentiated into myotubes. Upon transfection with either LNA-SSO 51.1c or AO51, immunofluorescence analysis reveals the presence of restored dystrophin localized to the plasma membrane (Fig 5). Thus, as previously shown for AO51, LNA-SSO 51.1c is most likely active in rescuing dystrophin production in differentiated muscle.

In conclusion, we demonstrate that 16-mer LNA-modified SSOs targeting a unique sequence in *DMD* exon 51 that has been highly evolutionary conserved effectively induce exon skipping and restore synthesis of a truncated dystrophin protein isoform that localizes to the plasma membrane of patient-derived myotubes differentiated in culture.

## Discussion

The selection of an antisense oligonucleotide sequence capable of inducing exon skipping is difficult to predict. Although statistical modeling approaches have been used to derive algorithms that predict exon skipping efficacy [44], experimental screening of multiple oligonucleotides remains the most reliable strategy. Efficient exon skipping requires that the SSO binds tightly to the nascent pre-mRNA and prevents assembly of the spliceosome at the target splice sites. Accordingly, LNA-modified SSOs targeting either the acceptor or donor splice sites of the *DMD* exon 58 were shown to be effective in inducing exon skipping. In addition, SSOs targeting exonic regions have also been reported to induce exon skipping, most probably through interference with RNA structures and/or binding of regulatory proteins that are critical for spliceosome assembly and splicing [29,38,45,46]. Because sequences important for splicing control tend to be evolutionary conserved [47,48], we designed antisense oligonucleotides targeting the two most conserved unique sequences in *DMD* exon 51 and we show that both are effective in inducing exon skipping. Thus, evolutionary conservation is probably a good criterion for selection of SSOs.

The results reported in this study using oligonucleotides with 60% of LNA-modified bases are in good agreement with previous observations indicating that 15-mer SSOs containing 8 LNA units showed high exon skipping activity [29]. SSOs fully modified with LNA were previously reported to have lower skipping activity, presumably because they form stable self-dimers or fail to recycle after splicing inhibition [20,29,49,50].

Our results further reveal that 16-mer LNA-SSOs effectively induce skipping of dystrophin exon 51. These LNA-SSOs are significantly shorter than drisapersen (24-mer) and eteplirsen (30-mers), which have been used in clinical trials. Shorter oligonucleotides are advantageous because they can bind more specifically [51]. This is because longer oligonucleotides have a higher chance of binding to off-target sequences containing a few mismatches [51]. LNA oligonucleotides as short as 8-mers were shown to bind and inhibit microRNA activity without off-target effects [52], and 7-mer LNA-SSOs were reported to modulate splicing [29]. However, for each target exon there are optimal sequences, lengths and number of LNA modifications. For example, a 13-mer LNA-SSO induced skipping of *DMD* exon 58 with higher efficacy than a 7-mer, and SSOs longer than 15 nucleotides showed reduced activity [29].

In addition to assessing the effect of SSOs by RT-PCR, we demonstrate that 16-mer LNA-modified SSOs effectively restore synthesis of a truncated dystrophin protein isoform that localizes to the plasma membrane of patient-derived myotubes differentiated in culture. Thus, our results strengthen the view that introducing LNA modifications in oligonucleotides allows for the use of shorter antisense sequences for therapeutic modulation of splicing.

## Supporting information

S1 Fig RT-PCR analysis of exon 51 skipping. DM8036 cells were transfected with each SSO at the indicated concentrations and analyzed 7 days after induction of myotube differentiation. Electrophoresis of PCR products in agarose gels shows non-skipped and skipped transcripts in independent biological experiments. Mock: mock transfection; -RT: no retrotranscriptase.
(TIF)

S2 Fig RT-PCR comparison of LNA-SSOs and AO51. DM8036 cells were transfected with each SSO at the indicated concentrations and analyzed 2 days after induction of myotube differentiation. Electrophoresis of PCR products in agarose gels shows non-skipped and skipped transcripts in independent biological experiments. Mock: mock transfection; -RT: no retrotranscriptase.
(TIF)

S3 Fig Effect of SSO transfection on myotube differentiation. DM8036 cells were either mock transfected or transfected with the indicated SSO at 50 nM. (A) Cells were observed by phase-contrast microscopy 3 and 5 days after induction of myotube differentiation. (B) Fusion index was calculated as the percentage of total nuclei in myotubes relative to the total number of nuclei. At least 200 nuclei were counted in each experiment. Error bars represent standard deviation (N = 3).
(TIF)
