# Supplementary figures and images for: Short (16-mer) locked nucleic acid splice-switching oligonucleotides restore dystrophin production in Duchenne Muscular Dystrophy myotubes
Source: PLoS One. 2017 Jul 24;12(7):e0181065. doi: 10.1371/journal.pone.0181065 (PMC5524367; doi:10.1371/journal.pone.0181065)

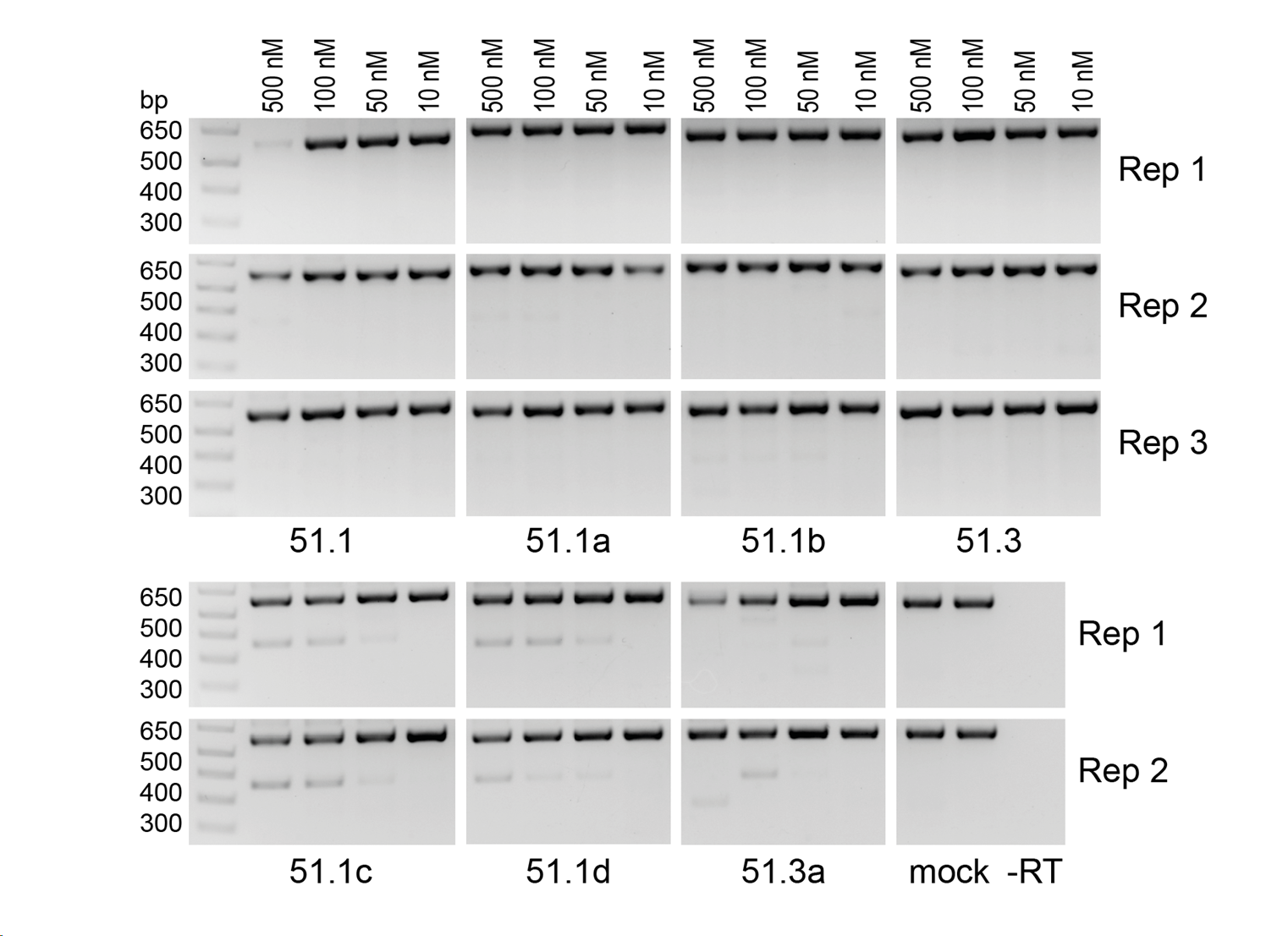

Supplement: S1 Fig — DM8036 cells were transfected with each SSO at the indicated concentrations and analyzed 7 days after induction of myotube differentiation. Electrophoresis of PCR products in agarose gels shows non-skipped and skipped transcripts in independent biological experiments. Mock: mock transfection; -RT: no retrotranscriptase. (TIF) [file pone.0181065.s001.tif]

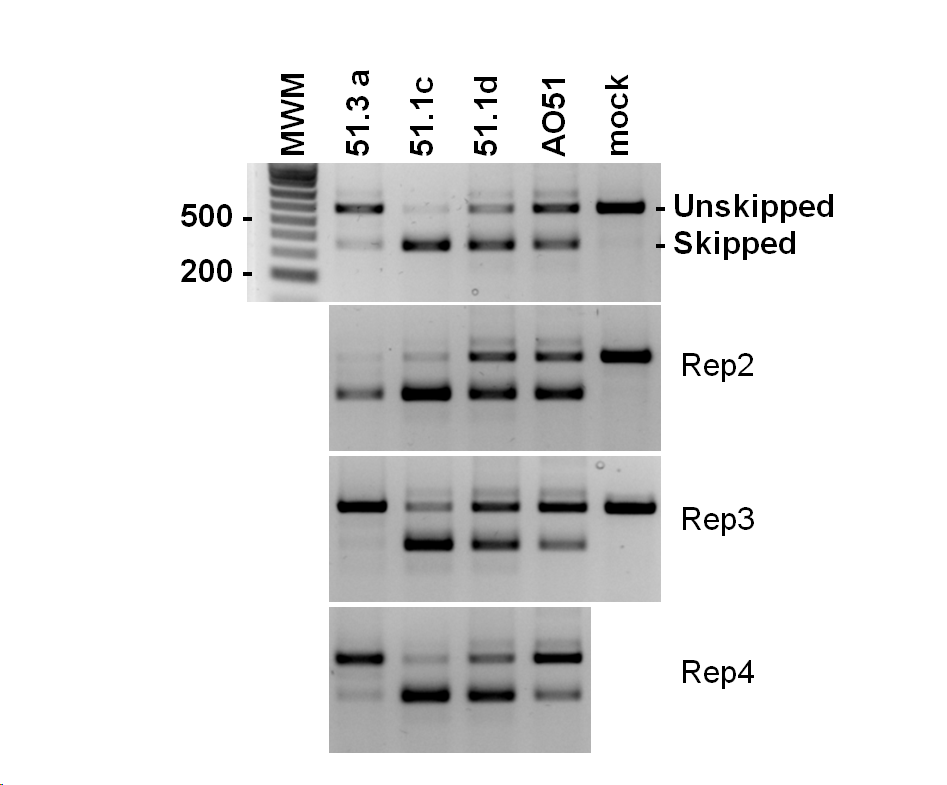

Supplement: S2 Fig — DM8036 cells were transfected with each SSO at the indicated concentrations and analyzed 2 days after induction of myotube differentiation. Electrophoresis of PCR products in agarose gels shows non-skipped and skipped transcripts in independent biological experiments. Mock: mock transfection; -RT: no retrotranscriptase. (TIF) [file pone.0181065.s002.tif]

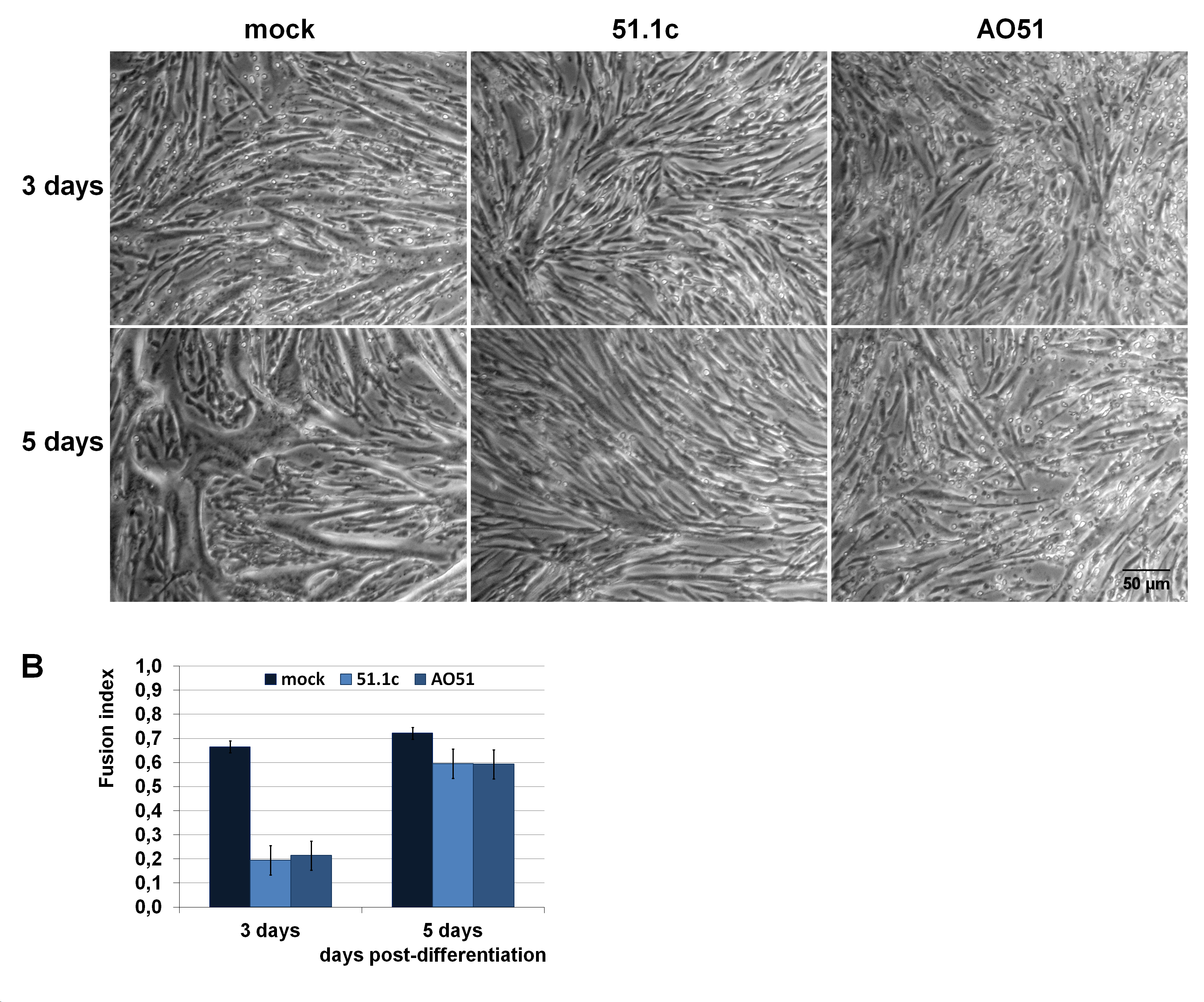

Supplement: S3 Fig — DM8036 cells were either mock transfected or transfected with the indicated SSO at 50 nM. (A) Cells were observed by phase-contrast microscopy 3 and 5 days after induction of myotube differentiation. (B) Fusion index was calculated as the percentage of total nuclei in myotubes relative to the total number of nuclei. At least 200 nuclei were counted in each experiment. Error bars represent standard deviation (N = 3). (TIF) [file pone.0181065.s003.tif]
